# Co-occurrence of IBS and symptoms of anxiety or depression, among Norwegian twins, is influenced by both heredity and intrauterine growth

**DOI:** 10.1186/s12876-015-0237-y

**Published:** 2015-02-05

**Authors:** May-Bente Bengtson, Geir Aamodt, Morten H Vatn, Jennifer R Harris

**Affiliations:** 1 Medical Department, Vestfold Hospital Trust, PO Box 2168, 3103 Tønsberg, Norway; 2 Norwegian University of Life Sciences, PO Box 5003, 1432 Ås, Norway; 3 EpiGen-Institute, Faculty Division Akershus University Hospital, University of Oslo, Oslo, Norway; 4 Division of Epidemiology, The Norwegian Institute of Public Health, Oslo, Norway

**Keywords:** IBS, Anxiety, Depression, Comorbidity, Birth weight, Norwegian twin registry (NTR)

## Abstract

**Background:**

Environmental and genetic factors contribute to variation in irritable bowel syndrome (IBS), anxiety and depression. Comorbidity between these disorders is high. A previous investigation of our population-based twin cohort revealed that low birth weight increased the risk for development of IBS, with environmental influences in utero as the most relevant contributing factor. We hypothesise that both intrauterine and genetic factors influence the co-occurrence of IBS and symptoms of anxiety and depression.

**Methods:**

A postal questionnaire sent to 12700 Norwegian twins born between 1967 and 1979 comprised a checklist of 31 illnesses and symptoms, including IBS and symptoms of anxiety and depression. The influence of genetic factors and intrauterine growth on comorbidity between these disorders were analysed in the full sample and compared to those based on only monozygotic (MZ) twin pairs discordant for IBS (95 pairs) in birth weight group < 2500 g and ≥ 2500 g.

**Results:**

In the co-twin analyses restricted growth (birth weight < 2500 g) was significantly associated with anxiety and depression (average birth weight difference of 181.0 g (p <0.0001) and 249.9 g (p < 0.0001), respectively).

The analysis of the full sample revealed that IBS was significantly associated with symptoms of anxiety (adjusted OR = 2.5, 95% CI: 1.9, 3.3) and depression (adjusted OR = 2.3. 95% CI: 1.8, 3.0). Analyses of MZ pairs discordant for IBS indicated significant associations between IBS and symptoms of anxiety (OR = 3.7, 95% CI: 1.3, 10.5) and between IBS and symptoms of depression (OR = 4.2, 95% CI: 1.7, 9.9) only in the birth weight group below 2500 g.

**Conclusion:**

Our findings suggest that genetic factors partly explain the association between IBS and symptoms of anxiety and depression. In the low range of birth weight (<2500 g), restricted fetal growth seems to be a common contributing factor to the co-occurrence between these disorders.

## Background

Irritable bowel syndrome (IBS) is characterized by abdominal pain and abnormal bowel pattern affecting approximately 10–20% of the general population [1,2]. The pathogenesis remains unclear, but accepted mechanisms involve an interaction between triggering environmental factors and abnormal gastrointestinal motility and disturbed visceral sensory perception [3-7]. Gene-environmental interactions are also important in the development of IBS, at least for sub-groups of IBS patients [7-10].

Multiple investigations conducted during the past two decades implicate genetic factors in the development of IBS. Family studies reveal that the frequency of self-reported IBS was significantly greater among those who reported having a first-degree relative with bowel problems than among those whose spouse had IBS [9]. Results from our population-based Norwegian twin study [8] concur with twin-based findings from other countries [10,11] and highlight the importance of genetic influences on IBS with significantly greater concordance rates among monozygotic (MZ) compared to dizygotic (DZ) pairs. Our earlier twin study [8] also demonstrated an association between low birth weight (LBW), < 2500 g, and development of IBS later in life; moreover, birth weight below 1500 g was associated with early onset of symptoms.

IBS patients report more frequent emotional distress and depression than controls, and IBS symptoms are often exacerbated during periods of stressful events [12]. Furthermore, anxiety and depression contribute to poor outcomes in IBS [13,14]. Numerous investigations have reported comorbidity between IBS and stress-related psychiatric disorders as high as 30–50% [14-18]. However, studies of the causal relation between these disorders are sparse and provide contradictory results [19-21].

In the present study we hypothesise that restricted intrauterine growth and familial factors influence the co-occurrence of IBS and mental distress. Familial factors comprise genetic factors and/or early familial influences associated with being raised as twins in the same household.

We used a case–control and co-twin control design in combination to assess whether co-morbidity between IBS and the mental health outcomes is due to within pair familial factors shared by the twins or due to a causal link between them. Furthermore, we expanded this test of the sources of comorbidity to explore the influence of restricted fetal growth by stratifying the co-twin control study into two birth weight groups, < 2500 g and ≥ 2500 g.

## Methods

The data are based on a population-based sample of twins [22,23] identified from the Norwegian national birth registry, which was established in 1967. Postal questionnaires were sent in 1998 to 12700 twins born between 1967 and 1979.

The final sample included 8045 twins (63% response rate), 3334 twin pairs and 1377 single responders. Of 3334 twin pairs, 138 pairs were excluded due to incomplete data. The final sample included 504 male monozygotic (MZM), 379 male dizygotic (DZM), 746 female monozygotic (MZF), 635 female dizygotic (DZF), and 932 dizygotic unlike pairs (DZU). Among these, there were 321 twin pairs for which at least one of the twins reported a positive history of IBS.

Initial screening for potential cases was conducted using self-reported questionnaire responses. The questionnaire included a checklist for 31 illnesses and symptoms, including IBS. The question was phrased as: Do you have, or have you ever had irritable bowel syndrome (diarrhoea, constipation, and painful abdominal distension). Two other questions on gastrointestinal diseases were asked: one question inquired about symptoms consistent with dyspepsia, where we used the phrase; recurrent upper abdominal pain, and another concerning inflammatory bowel disease (IBD), where we used the terms Crohn’s disease and ulcerative colitis. A diagnosis of IBD was confirmed through the patient’s doctor. Age at onset was asked, and if symptoms were not currently present, the subject was asked to record the time of the last event. Age at onset and cessation of symptoms were coded as continuous variables. Thirteen twins checked the box for both IBS and IBD. Seven of these responded to an invitation for a study of IBD, and consented to a phone interview. Four of these patients appeared to have IBS, and not IBD, and three had IBD, which was confirmed by journal records. The twins with IBD (three twins) and the none-responders (six twins) were excluded, which resulted in data from 312 twin pairs available for the analyses.

Items pertaining to sleep disorder and somatic disorders, such as frequent headache, long-lasting muscle pain and diabetes were also included in the questionnaire. Symptoms of anxiety and depression were assessed using five questions from the Symptom Check List (SCL-5) anxiety and depression subscales that were also included in the questionnaire. This short version has been shown to correlate 0.92 with the full scale score list of Hopkin’s (HSCL-25) anxiety and depression score [24], 10 items for anxiety and 15 items for depression.

The short form version included two items of anxiety; 1) feeling fearful, 2) nervousness or shakiness inside, and 3 items of depression; 1) feeling hopeless about the future, 2) feeling blue, 3) worrying too much about things. The participants were asked to classify the symptoms according to a four-point scale with anchors 1 (Not at all) to 4 (Extremely).

Values for the symptoms of anxiety and depression were calculated by summing the scores for each item belonging to the respective subscale and computing mean values. Mean values ≥ 2 were used as cut of points for symptoms of anxiety and depression [25].

Marital status included; 1) unmarried/not live-in partner, 2) married/live-in partner, 3) widow/widower, 4) divorced/separated. Educational level was split in two variables; completed and planned education, because the participants were aged between 19 and 31 years. The education level included: 1) high school or secondary school plus two or three years additional training or economic education, 2) college or university up to 4 years, 3) master degree or PhD.

Data on birth weight and gestational age were obtained from the Medical Birth Registry of Norway. Classification of zygosity was based on responses to seven questionnaire items, which have been verified in a previous study of Norwegian twins to correctly categorize zygosity with 97% accuracy [22]. These classifications were further verified through an update of The Norwegian Institute of Public Health twin program of research [23] using twenty-four microsatellite markers which were genotyped on 676 of the like-sex pairs in the sample. Results from these markers were used as dependent variables in a discriminant analysis with the questionnaire items as independent variables. Seventeen of these pairs (2.5%) were misclassified by the questionnaire data and zygosity classifications were corrected. From these data, we estimated that, in our entire sample, zygosity misclassification occurred in 1% of pairs, a rate unlikely to substantially bias results.

### Analyses

Probandwise concordance was calculated by zygosity for anxiety and depression. This concordance rate is defined as the ratio of twice the number of concordant pairs, divided by twice the number of concordant pairs plus the number of discordant pairs. Differences in concordance rates between MZ and DZ pairs were tested using χ^2^ statistics.

To indicate the level of comorbidity between depression and anxiety, the ĸ statistics were measured, and reported with 95% confidence interval (CI). A value of 1.00 indicate perfect agreement, whereas 0.00 only agreement by chance.

Paired-t tests were used to analyse the associations between LBW with symptoms of anxiety and depression. To control for genetic factors, which may contribute to low birth weight as well as to anxiety and depression, two separate analyses were conducted using only data from MZ pairs discordant for anxiety or depression, respectively. The data were stratified into two groups according to the birth weight of the affected twin, ≥ 2500 g or < 2500 g, and differences in mean birth weight between twins with and without symptoms of anxiety or depression were estimated. To increase statistical power, the birth weight group < 2500 g included pairs where both twins were below 2500 g and twin pairs where only the affected twin had a birth weight below 2500 g. Separate logistic regression models were fitted for anxiety and depression to analyse potential confounding effects of marital status and education. Birth weight, split into quartiles, was included in these models.

To explore if age at onset of symptoms of IBS was a risk factor for the association between IBS and depression or anxiety, the data from twins with IBS were stratified into two groups, representing those older and younger than 15 years at age of IBS onset. Age at survey was grouped into four categories each representing 3-year intervals.

### Co-occurrence of IBS with symptoms of anxiety or depression

Logistic regression models were used to analyse the co-occurrence of IBS with symptoms of anxiety and depression. Separate models were analysed for the association between IBS with each mental health outcome. Odds ratios (OR) with 95% confidence intervals (CI) were calculated adjusted for gender, age at the time of survey and twin relatedness (GEE), using SPSS 20 in the full twin sample. ORs in the co-twin control analyses were estimated using logistic regression with twin pairs as the unit of analysis.

To assess possible effect measure modification, interactions between anxiety and gender and between gender and depression were included in the model. Interaction terms which were significant for the associations IBS-anxiety or IBS-depression, were included in the models because risk of anxiety and depression could be different for the men and women belonging to the same age groups.

The associations between IBS and symptoms of anxiety or depression were first tested in the full twin sample (case–control design), and further analysed using only data from IBS-discordant MZ twins (co-twin control design). Adjusting for twin relatedness in the analyses in the full twin sample supports generalisation to the background population.

This methodology was described previously by Wojczynski et al. [21], and is based on the assumption that resemblance between MZ twins, who are genetically identical, may also stem from shared early environmental factors important for the disorder of interest such as parental rearing, childhood trauma, same housing, pets, similar diet and same infectious diseases, etc.

Therefore, if an association is found in the case–control design study but not in the co-twin control design, this indicates that the comorbidity depends partly on genetic and/or family environment influences. However, if an association is found between IBS and the mental health measures in both the case–control and the co-twin control design, this would suggest a causal link between these health outcomes [21].

We expanded this test of the sources of comorbidity to explore the influence of restricted fetal growth by stratifying the co-twin control study into two birth weight groups, < 2500 g and ≥ 2500 g. If an association of IBS with the mental health outcomes is demonstrated only in the lowest birth weight group this would suggest that comorbidity arises from a causal biological mechanism due to restricted fetal growth.

### Ethical approval

This study was evaluated and approved by the Norwegian Scientific Ethics Committees and by the Norwegian Data Protection Agency. Our ethics review approved that the return of the questionnaire by the twins, meant that they consented. In the invitation letter, dispatched with the questionnaire, we explained how the data would be used and that the study was voluntary.

## Results

The lifetime prevalence of IBS in this twin cohort has been previously reported to be 5.3%, with a female predominance of 76.5%. Furthermore, the majority of cases (75.5%) reported ongoing IBS symptoms at the time of survey [8]. The present study revealed that 13 and 15.5% of the twins reported ongoing symptoms of anxiety and depression, respectively. Females accounted for the majority of those reporting symptoms of anxiety (73.5%) and depression (68.3%).

The number of twin pairs and cases by zygosity are listed in Table 1 for each health condition. The concordance rates are presented in Table 2. Concordance was significantly greater among MZ than DZ pairs for symptoms of anxiety (p = 0.03), symptoms of depression (p < 0.0001) and IBS (p = 0.011).

**Table 1:** **Number of twin pairs and cases for each disorder by zygosity in a Norwegian population-based cohort**

Groups	Pairs	Cases of irritable bowel syndrome	Cases of depression	Cases of anxiety
Monozygotic	1250	123	336	298
Dizygotic	1946	213	619	532
Total	3196	336	985	830

**Table 2:** **Concordance rates and probandwise concordance rates for irritable bowel syndrome (IBS), depression and anxiety among monozygotic (MZ) and dizygotic (DZ) twins**

		MZ	DZ	Total no pairs
IBS	Concordant pairs	14	10	24
	Discordant pairs	95	193	288
	Probandwise concordance rate	0.23	0.09*	
Depression	Concordant pairs	67	82	149
	Discordant pairs	232	455	687
	Probandwise concordance rate	0.22	0.15*	
Anxiety	Concordant pairs	56	55	111
	Discordant pairs	186	422	608
	Probandwise concordance rate	0.23	0.12*	

*Significant higher concordance rate among MZ compared to DZ twins.

Somatic comorbidity including factors such as sleep disorder, frequent headache, long-lasting muscles pain and dyspepsia were more common among cases of IBS (Table 3) and among those reporting symptoms of anxiety and depression (Table 4), compared to controls. There was a high level of comorbidity between symptoms of anxiety and depression (κ = 0.49, 95% CI: 0.45, 0.52) (Table 4). Age at onset of IBS symptoms did not influence the associations between IBS and symptoms of anxiety (OR = 1.2, 95% CI: 0.6, 2.5) or depression (OR = 1.2, 95% CI: 0.6, 2.1).

**Table 3:** **Somatic – psychiatric comorbidity among irritable bowel syndrome (IBS) cases and controls**

	IBS case–control population (N = 6374)		
	Cases N (%)	Controls N (%)	OR (95% CI)
	(N = 336)	(N = 6038)	
Female	258 (76.8)	3423 (56.7)	2.5 (1.9, 3.3)
Sleep disorder	87 (25.9)	420 (7)	4.7 (3.6, 6.1)
Frequent headache	87 (25.9)	626 (10.4)	3.0 (2.3, 3.9)
Long-lasting muscles pain	48 (14.3)	219 (3.6)	4.4 (3.2, 6.2)
Dyspepsia	69 (20.5)	175 (2.9)	8.7 (6.4, 11.7)
Depression	102 (30.4)	569 (9.4)	2.8 (2.2, 3.7)
Anxiety	95 (28.3)	333 (5.5)	3.1 (2.3, 4.3)

**Table 4:** **Somatic comorbidity among twins with depression and anxiety in the Norwegian twin registry**

	Case–control population (N = 6374)					
	Depression			Anxiety		
	Cases N (%)	Controls N (%)	OR (95% CI)	Cases N (%)	Controls N (%)	OR (95% CI)
	N = 985	N = 5389		N = 830	N = 5544	
Female	673 (68.3)	3008 (55.8)	1.7 (1.5, 1.9)	610 (73.5)	3071 (55.4)	2.2 (1.9, 2.6)
Sleep disorder	220 (22.3)	287 (5.3)	5.1 (4.2, 6.2)	205 (24.7)	302 (5.4)	5.7 (4.7, 6.9)
Frequent headache	179 (18.2)	534 (9.9)	2.0 (1.7, 2.4)	176 (21.2)	537 (9.7)	2.5 (2.1, 3.0)
Long-lasting muscles pain	82 (8.3)	185 (3.4)	2.5 (1.9, 3.3)	83 (8.9)	184 (3.3)	3.2 (2.5, 4.2)
Dyspepsia	71 (7.2)	173 (3.2)	2.2 (1.8, 3.1)	74 (8.9)	170 (3.1)	3.1 (2.3, 4.1)
Diabetes	10 (1)	38(0.7)	1.4 (0.7, 2.9)	10 (1.2)	38 (0.7)	1.8 (0.9, 3.6)
Irritable bowel syndrome	77(11.9)	259 (4.5)	2.8 (2.1, 3.7)	95 (11.4)	2841(4.3)	2.8 (2.2, 3.7)
Depression				510 (61.4)	475 (8.6)	17.0 (14.3, 20.1)
Anxiety	510 (51.8)	320 (5.9)	17.0 (14.3, 20.1)			

### Effect of birth weight using a co-twin design approach

Among the MZ twins, 186 pairs were discordant for the measure of anxiety and 232 pairs were discordant for the measure of depression. In these pairs, low birth weight (below 2500 g) occurred for 77/186 of the anxiety discordant pairs and 100/232 of the depression discordant pairs. The affected twins in the low birth weight group weighed significantly less at birth compared to their unaffected co-twins with average birth weight differences of 181.03 g (p <0.0001) for symptoms of anxiety and 249.95 g (p < 0.0001) for symptoms of depression. Twins with and without anxiety and depression in the low birth weight group, did not differ regarding complete or planned education status. The risk of depression, but not anxiety, was significantly lower for married compared to unmarried participants (OR = 0.4, 95% CI: 0.2, 0.8 and OR = 0.8, 95% CI: 0.4, 1.6, respectively). The odds for depression, adjusted for marital status, was 2.7 times higher (95% CI: 1.2, 5.9) for those in the lower quartile group of birth weight compared to those in the upper quartile group.

### Case–control model

Of the 336 twins who reported IBS, 95 (28.3%) and 102 (30.4%) also reported symptoms of anxiety and depression, respectively. The case–control analyses revealed a significant association between IBS and symptoms of anxiety and between IBS and symptoms of depression (crude OR = 2.8, 95% CI: 2.2, 3.7, and crude OR = 2.5. 95% CI: 1.9, 3.3, respectively). Female gender, but not age at survey, appeared to be an independent risk factor for IBS in the models. The associations between IBS and symptoms of anxiety or depression were slightly attenuated when adjusted for gender (OR = 2.5, 95% CI: 1.9, 3.3, and OR = 2.3, 95% CI: 1.8, 3.0, respectively).

Comorbid sleeping disorder was significantly higher among IBS cases with symptoms of anxiety and depression compared to IBS cases without these disorders (41/95, 43.2%, OR = 2.3, 95% CI: 1.6, 3.2 and 41/102, 40.2%, OR = 2.0, 95% CI: 1.4, 2.9). Cases of IBS with anxiety (20/95, 21.1%, OR = 1.8, 95% CI: 1.1, 3.1) were more likely to have comorbid long-lasting muscle pain, but depression did not reach significance (20/102, 19.6%, OR = 1.6, 95% CI: 0.9, 2.8). Comorbid frequent headache did not show a similar trend.

### Co-twin control analyses: MZ twins discordant for IBS

The analyses of data from MZ twins discordant for IBS (95 pairs) revealed that the risk of comorbid symptoms of anxiety (24/95, 25.3%) (OR = 2.2, 95% CI: 1.1, 4.2) and depression (28/95, 29.5%) (OR = 1.9, 95% CI: 0.9, 3.7) was higher among twins with IBS compared to those without IBS, but did not reach significance for depression (p = 0.055). Sleeping disorder (24/95, 25.3%) (OR = 2.9, 95% CI: 1.3, 6.4) and long-lasting muscle pain (16/95, 16.8%) (OR = 9.4, 95% CI: 2.1, 42.2) were significantly associated with IBS, but frequent headache was not associated with IBS (27/95, 28.4%) (OR = 1.5, 95% CI: 0.8, 2.9).

### Co-twin control analyses: MZ twins discordant for IBS by birth weight group

**Figure 1:**
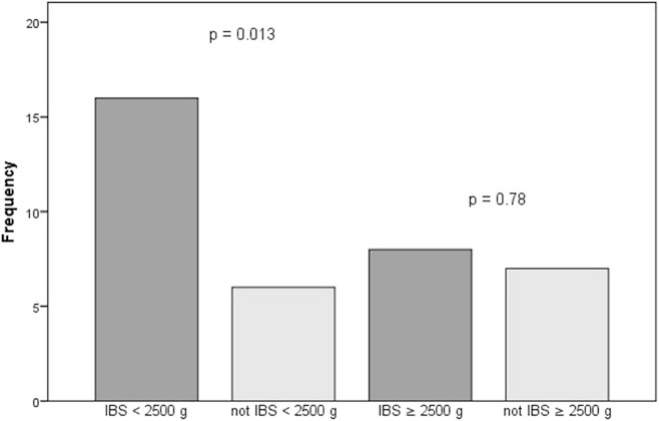
**Symptoms of anxiety among MZ twins discordant for IBS.** The assoiation between IBS and symptoms of anxiety among MZ twins was demonstrated only in the birth weight groups < 2500 g, indicating restricted fetal growth as a common contributing factor in the low range of birth weight.

**Figure 2:**
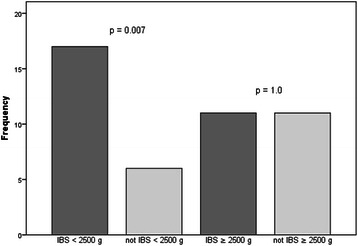
**Symptoms of depression among MZ twins discordant for IBS.** The association between IBS and symptoms of depression was demonstrated only in the birth weight group < 2500 g, indicating restricted fetal growth as a common contributing factor in the low range of birth weight.

Data from MZ twin pairs discordant for IBS were stratified into birth weight groups defined as < 2500 g or ≥ 2500 g. Significant associations were found between IBS and symptoms of anxiety (16/41, 39.0%) (OR =3.7, 95% CI: 1.3, 10.5) (Figure 1), and between IBS and symptoms of depression (17/41, 41.5%) (OR = 4.2, 95% CI: 1.7, 9.9) in the weight group < 2500 g (41 pairs) (Figure 2). Among the twins within normal range of birth weight, ≥ 2500 g (54 pairs), there was no association between IBS and symptoms of anxiety (8/54, 14.8%) (OR = 1.2, 95% CI: 0.4, 3.5) or between IBS and symptoms of depression (11/54, 20.4%) (OR = 1.0, 95% CI: 0.4, 3.5). The risk for comorbid symptoms of anxiety (OR = 2.6, 95% CI: 1.3, 5.6) and depression (OR = 2.0, 95% CI: 1.1, 3.9) among twins with IBS weighing less than 2500 g at birth was twice as high compared to those with IBS and weighing ≥ 2500 g.

## Discussion

This study demonstrated that both familial and intrauterine factors influence the co-occurrence of IBS and symptoms of anxiety and depression in a Norwegian population-based twin cohort. To examine the nature of the comorbidity between these disorders, associations between IBS and symptoms of anxiety and depression were first tested in the full twin sample (case–control), and then further analysed using data from MZ twin pairs discordant for IBS. The results from these analyses were compared to help elucidate the importance of familial factors; genetic factors and/or family environment, on the comorbidity of these disorders. Further analyses using only discordant MZ twin pairs (co-twin control design) stratified into two birth weight groups, < 2500 g and ≥ 2500 g explored the significance of intrauterine conditions as a common risk factor for comorbidity of IBS with the mental health outcomes. Our analyses confirmed associations between IBS and symptoms of anxiety and depression (OR = 2.5, 95% CI: 1.9, 3.3, OR = 2.3, 95% CI: 1.8, 3.0, respectively) in the case–control study as well as in the co-twin design study. These findings are in agreement with the results of Wojczynski et al., who introduced this novel twin study design in a population-based Swedish twin cohort [21]. They consequently concluded that comorbidity between IBS and depression could not be explained by genetic or family environmental factor, but rather by a possible causal link between these disorders.

However, in the present study, we were able to demonstrate that both genetic and environmental mechanisms contribute to the comorbidity between mental disorders and IBS, by stratifying the co-twin control study into two birth weight groups, < 2500 g and ≥ 2500 g.

Our analyses revealed significant comorbidity between IBS and symptoms of anxiety and depression only in the birth weight group < 2500 g (OR =3.7, 95% CI: 1.3, 10.5, OR = 4.2, 95% CI: 1.7, 9.9, respectively). In normal range of birth weight (≥2500 g), we did not find any association between IBS and mental health conditions, in contrast to the case–control findings, indicating that comorbidity of IBS with symptoms of anxiety and depression could partly be explained by genetic or family environment influence. Furthermore, results from these analyses help to disentangle effects due to restricted fetal growth as a significant common contributor to the co-occurrence of these disorders in the low range of birth weight scale (<2500 g).

Genetic modelling based on this twin cohort have earlier demonstrated that shared family environment did not contribute to development of either IBS or symptoms of anxiety and depression [8,26], suggesting that genetic factors account for the main source of resemblance between MZ twins when it comes to these disorders.

The influence of restricted birth weight for development of mental health conditions was confirmed by the paired t-tests in the birth weight group < 2500 g. These analyses yielded a significant weight difference of 181.0 g (p < 0.0001) and 249.9 g (p < 0.0001) favouring the twin without symptoms of anxiety and depression, respectively. Marital status and education level did not modify the association between these disorders and restricted intrauterine growth. Lower birth weight in affected twins, meaning restricted growth, indicates that environmental factor during fetal life play an important role for development of symptoms of anxiety and depression later in life. These results are in line with other investigations revealing that low birth weight (LBW), defined as birth weight < 2500 g, increases the risk for behavioural, emotional, and learning difficulties as well as poor cognitive function in school-age children [27-29]. The link between LBW and development of mental health conditions later in life might be explained by long-term dysfunction of the Hypothalamic-Pituitary- Adrenal (HPA) -axis induced in fetal life by programming. Fetal programming is a process whereby a stimulus or an insult in a critical period during fetal life results in permanent change of structure or physiology of the organism [30]. Both physical and psychological stressors contribute to hypothalamic release of corticotropin-releasing factor (CRF) [31,32], which stimulates release of the stress hormones adrenocorticotropin hormone (ACTH) from the pituitary and cortisol from the adrenal glands. Measurements of basal cortisol and ACTH, as well as stimulated cortisol and ACTH are the most common approach to evaluate the function of the HPA-axis [33]. Exposure to intrauterine stress, measured by LBW, may increase the vulnerability to subsequent normative stress later in life by changing the function of the HPA-axis [34].

Changed basal levels of cortisol [35] and ACTH have also been demonstrated among patients with IBS. Dysfunctional HPA-axis involves the pathogenesis of IBS by contributing to visceral hypersensitivity [5] and altered bowel motility [6]. The association between restricted birth weight and symptoms of anxiety or depression in the present study, and between IBS and restricted birth weight showed in our previous study [8], might suggest dysfunctional HPA-axis as a common risk factor for these disorders, in the LBW group. Moreover, the comorbidity between IBS and symptoms of anxiety or depression was confirmed only in the LBW group, suggesting that the same mechanism contributes to the occurrence between these disorders.

The analyses in the full sample of twins revealed significant associations between IBS and somatic disorders like sleeping disorder, frequent headache and long-lasting muscle pain. Furthermore, IBS cases with symptoms of anxiety and depression had an even higher risk for comorbid sleeping disorder and long-lasting muscle pain compared to IBS case without these comorbid disorders, in line with the results in the investigation of Lembo et al. [19]. In the co-twin design study similar associations were found between IBS and sleeping disorder and long-lasting muscle pain, indicating that these somatic disorders share common biological pathway as IBS. Sleeping disorder and long-lasting muscle pain, have been linked to hypervigilance to bodily symptoms, and closely associated with IBS [36]. Several twin and family investigations revealed that genetic factors contribute to the development of IBS [9,10] and account for a substantial part the variability of psychiatric disorders [37,38]. Furthermore, studies have demonstrated shared genetic factors between depression and anxiety disorder [39].

The concordance rate among MZ twins for anxiety (p = 0.03) and depression (p < 0.0001) in the present study, and for IBS (p = 0.011) demonstrated in the same twin cohort previously were significantly higher than among DZ twins [8]. Moreover, there was a large overlap between symptoms of anxiety and depression, with a kappa value of 0.49 (p <0.0001).

Recent investigations have shown that IBS and mental disorder share common genetic pathways like the serotonergic pathway and the corticotrophin releasing system [40].

We demonstrated comorbid symptoms of anxiety and depression among approximately 30% of the twins reporting symptoms of IBS in the full sample, and almost similar associations among MZ twins discordant for IBS. Furthermore, the risk of comorbid symptoms of anxiety (39.0%) and depression (41.5%) was twice as high in the birth weight group < 2500 g compared to the group ≥ 2500 g, suggesting a differentiation in the pathogenesis of sub-groups of IBS according to birth weight groups.

The estimated lifetime prevalence of IBS the study of Wojczynski et al. (2.06%) [21] was less than half the prevalence of IBS in the present study (5.3%), and probably reflects the difference of IBS definitions used in the studies. Wojczynski et al. explained that their use of an adapted version of the Rome criteria probably included mostly diarrhoea-predominant IBS, which usually represents less than a third of an IBS population [14].

The notably lower prevalence of IBS (2.06%), in conjunction with a higher lifetime prevalence of depression (21.5%) reported in the study of Wojczynski might partially explain why comorbidity between depression and IBS was much higher (45%), than we report (30.4%). The prevalence of IBS in our twin cohort (5.3%) is lower compared to the prevalence found in a population-based study in Norway from general practice (8.4%), which included approximately 11 000 inhabitants aged between 30 to 75 years [14]. The questionnaire used to determine IBS symptoms in that study [14] was based on the Rome II criteria in contrast to our study. The twins in our study seem to be familiar with the term irritable bowel syndrome, possibly as a result of medically based diagnosis, and responded to the symptoms, constipation, diarrhoea and painful abdominal distension, which all are important components of IBS. Furthermore, the typical chronic course of intermittent symptoms of IBS has been demonstrated earlier in the same twin sample as the present study [8].

In spite of lower lifetime prevalence of IBS in the Norwegian twin cohort compared to the study in general practice [14], both investigations demonstrated a 3- to 5-fold increased risk of comorbid disorders like depression, anxiety, skeletal and pain related disorder (Table 3). And even more important, Vandvik et al. [14] showed a prevalence of comorbid mood disorder of 25% among IBS consulters as well as IBS non-consulters. If the IBS diagnosis in the present study was mainly restricted to IBS consulters, comorbidity of symptoms of anxiety and depression is comparable with the findings in the background population.

Vanvik et al. [14] measured anxiety and depression symptom with the Hopkins’ Symptoms Checklist-10 with cut-off point of ≥ 1.85, while the present study included 5 instead of 10 symptoms in the list for anxiety and depression (Hopkins’ Symptoms Checklist-5) with cut-off point ≥2, in line with the recommendation as valid predictor of mood disorder [25].

Only gender (male), which questionnaire was received first, and low birth weight were associated with decreased possibility of participating in our study. Responses to the questionnaire were received from a larger percentage of females (70%) than males (56%), which might part explain the predominance of IBS among females (7.0%) compared to males (2.9%). The difference in birth weight between our twin cohort and non-responders was 29 g (95% CI: 9.3, 49.4, p = 0.004) and could lead to a slightly underestimation of odds for IBS as well as anxiety and depression, and therefore unlikely to influence the comorbidity between these disorders.

## Conclusions

The present twin study revealed that both genetic factors and intrauterine factors influence the associations between IBS, anxiety and depression.

The associations between IBS and mental disorders were demonstrated in the case–control study as well as in the co-twin analyses, but only among twins with low birth weight (<2500 g). These findings suggest, for the first time to our knowledge that restricted fetal growth seems to be a common contributing factor to the comorbidity between IBS, anxiety and depression in the low range of birth weight. Furthermore, the associations found in the case–control study did not appear in the co-twin analyses among twins in normal range of birth weight (≥2500 g) suggesting that genetic factors in part could explained the associations between these disorders. Our results might contribute to the understanding of the underlying mechanisms of IBS and the sub-classification of IBS.

## Abbreviations

ACTH: Adrenocorticotropin hormone
CI: Confidence interval
CRF: Cortico-releasing factor
DZ: Dizygotic
DZF: Dizygotic female
DZM: Dizygotic male
DZU: Dizygotic unlike pairs
GEE: Generalize estimating equation
HPA-axis: Hypothalamic-pituiary-adrenal axis
HSCL-25: Hopkin’s full scale score list
IBD: Inflammatory bowel disease
IBS: Irritable bowel syndrome
LBW: Low birth weigh
MZ: Monozygotic
MZF: Monozygotic female
MZM: Monozygotic male
NTR: Norwegian twin registry
OR: Odds ratio
SCL-5: Symptom Check List

## References

[1] Thompson WG, Irvine EJ, Pare P, Ferrazzi S, Rance L (2002). Functional gastrointestinal disorders in Canada: first population-based survey using Rome II criteria with suggestions for improving the questionnaire. Dig Dis Sci.

[2] Hungin AP, Whorwell PJ, Tack J, Mearin F (2003). The prevalence, patterns and impact of irritable bowel syndrome: an international survey of 40,000 subjects. Aliment Pharmacol Ther.

[3] Drossman DA, Camilleri M, Mayer EA, Whitehead WE (2002). AGA technical review on irritable bowel syndrome. Gastroenterology.

[4] Longstreth GF, Thompson WG, Chey WD, Houghton LA, Mearin F, Spiller RC (2006). Functional bowel disorders. Gastroenterology.

[5] Whitehead WE, Holtkotter B, Enck P, Hoelzl R, Holmes KD, Anthony J (1990). Tolerance for rectosigmoid distention in irritable bowel syndrome. Gastroenterology.

[6] Vassallo MJ, Camilleri M, Phillips SF, Steadman CJ, Talley NJ, Hanson RB (1992). Colonic tone and motility in patients with irritable bowel syndrome. Mayo Clin Proc.

[7] Spiller R, Aziz Q, Creed F, Emmanuel A, Houghton L, Hungin P (2007). Guidelines on the irritable bowel syndrome: mechanisms and practical management. Gut.

[8] Bengtson MB, Ronning T, Vatn MH, Harris JR (2006). Irritable bowel syndrome in twins: genes and environment. Gut.

[9] Kalantar JS, Locke GR, Zinsmeister AR, Beighley CM, Talley NJ (2003). Familial aggregation of irritable bowel syndrome: a prospective study. Gut.

[10] Levy RL, Jones KR, Whitehead WE, Feld SI, Talley NJ, Corey LA (2001). Irritable bowel syndrome in twins: heredity and social learning both contribute to etiology. Gastroenterology.

[11] Morris-Yates A, Talley NJ, Boyce PM, Nandurkar S, Andrews G (1998). Evidence of a genetic contribution to functional bowel disorder. Am J Gastroenterol.

[12] Blanchard EB, Lackner JM, Jaccard J, Rowell D, Carosella AM, Powell C (2008). The role of stress in symptom exacerbation among IBS patients. J Psychosom Res.

[13] Creed F, Ratcliffe J, Fernandes L, Palmer S, Rigby C, Tomenson B (2005). Outcome in severe irritable bowel syndrome with and without accompanying depressive, panic and neurasthenic disorders. Br J Psychiatry.

[14] Vandvik PO, Lydersen S, Farup PG (2006). Prevalence, comorbidity and impact of irritable bowel syndrome in Norway. Scand J Gastroenterol.

[15] Levy RL, Olden KW, Naliboff BD, Bradley LA, Francisconi C, Drossman DA (2006). Psychosocial aspects of the functional gastrointestinal disorders. Gastroenterology.

[16] Williams RE, Hartmann KE, Sandler RS, Miller WC, Steege JF (2004). Prevalence and characteristics of irritable bowel syndrome among women with chronic pelvic pain. Obstet Gynecol.

[17] Jarrett M, Heitkemper M, Cain KC, Burr RL, Hertig V (2000). Sleep disturbance influences gastrointestinal symptoms in women with irritable bowel syndrome. Dig Dis Sci.

[18] Whitehead WE, Palsson O, Jones KR (2002). Systematic review of the comorbidity of irritable bowel syndrome with other disorders: what are the causes and implications?. Gastroenterology.

[19] Lembo AJ, Zaman M, Krueger RF, Tomenson BM, Creed FH (2009). Psychiatric disorder, irritable bowel syndrome, and extra-intestinal symptoms in a population-based sample of twins. Am J Gastroenterol.

[20] Svedberg P, Johansson S, Wallander MA, Hamelin B, Pedersen NL (2002). Extra-intestinal manifestations associated with irritable bowel syndrome: a twin study. Aliment Pharmacol Ther.

[21] Wojczynski MK, North KE, Pedersen NL, Sullivan PF (2007). Irritable bowel syndrome: a co-twin control analysis. Am J Gastroenterol.

[22] Harris JR, Magnus P, Tambs K (2002). The Norwegian Institute of Public Health Twin Panel: a description of the sample and program of research. Twin Res.

[23] Harris JR, Magnus P, Tambs K (2006). The Norwegian Institute of Public Health twin program of research: an update. Twin Res Hum Genet.

[24] Tambs K, Moum T (1993). How well can a few questionnaire items indicate anxiety and depression?. Acta Psychiatr Scand.

[25] Strand BH, Dalgard OS, Tambs K, Rognerud M (2003). Measuring the mental health status of the Norwegian population: a comparison of the instruments SCL-25, SCL-10, SCL-5 and MHI-5 (SF-36). Nord J Psychiatry.

[26] Nes RB, Roysamb E, Reichborn-Kjennerud T, Harris JR, Tambs K (2007). Symptoms of anxiety and depression in young adults: genetic and environmental influences on stability and change. Twin Res Hum Genet.

[27] Gray RF, Indurkhya A, McCormick MC (2004). Prevalence, stability, and predictors of clinically significant behavior problems in low birth weight children at 3, 5, and 8 years of age. Pediatrics.

[28] Cheung YB, Ma S, Machin D, Karlberg J (2004). Birthweight and psychological distress in adult twins: a longitudinal study. Acta Paediatr.

[29] Saigal S, den Ouden L, Wolke D, Hoult L, Paneth N, Streiner DL (2003). School-age outcomes in children who were extremely low birth weight from four international population-based cohorts. Pediatrics.

[30] Barker DJ, Clark PM (1997). Fetal undernutrition and disease in later life. Rev Reprod.

[31] Elenkov IJ, Wilder RL, Chrousos GP, Vizi ES (2000). The sympathetic nerve–an integrative interface between two supersystems: the brain and the immune system. Pharmacol Rev.

[32] Fish EW, Shahrokh D, Bagot R, Caldji C, Bredy T, Szyf M (2004). Epigenetic programming of stress responses through variations in maternal care. Ann N Y Acad Sci.

[33] Jacobson L, Sapolsky R (1991). The role of the hippocampus in feedback regulation of the hypothalamic-pituitary-adrenocortical axis. Endocr Rev.

[34] Nomura Y, Wickramaratne PJ, Pilowsky DJ, Newcorn JH, Bruder-Costello B, Davey C (2007). Low birth weight and risk of affective disorders and selected medical illness in offspring at high and low risk for depression. Compr Psychiatry.

[35] Videlock EJ, Adeyemo M, Licudine A, Hirano M, Ohning G, Mayer M (2009). Childhood trauma is associated with hypothalamic-pituitary-adrenal axis responsiveness in irritable bowel syndrome. Gastroenterology.

[36] Sperber AD, Dekel R (2010). Irritable bowel syndrome and co-morbid gastrointestinal and extra-gastrointestinal functional syndromes. J Neurogastroenterol Motil.

[37] Sullivan PF, Neale MC, Kendler KS (2000). Genetic epidemiology of major depression: review and meta-analysis. Am J Psychiatry.

[38] Zubenko GS, Maher B, Hughes HB, Zubenko WN, Stiffler JS, Kaplan BB (2003). Genome-wide linkage survey for genetic loci that influence the development of depressive disorders in families with recurrent, early-onset, major depression. Am J Med Genet B Neuropsychiatr Genet.

[39] Middeldorp CM, Cath DC, Van Dyck R, Boomsma DI (2005). The co-morbidity of anxiety and depression in the perspective of genetic epidemiology. A review of twin and family studies. Psychol Med.

[40] Saito YA (2011). The role of genetics in IBS. Gastroenterol Clin North Am.

